# Ligamentum teres injuries - an observational study of a proposed new arthroscopic classification

**DOI:** 10.1093/jhps/hnv045

**Published:** 2015-07-01

**Authors:** Antonio Porthos Salas, John M. O’Donnell

**Affiliations:** 1. Hip Arthroscopy Mexico, Swiss Hospital, Uxmal 106-1, San Pedro Garza García 66290, México; 2. Hip Arthroscopy Australia, Melbourne, Australia; 3. Saint Vincent’s Hospital, Melbourne, Australia

## Abstract

Ligamentum teres (LT) Injuries or tears have been said to be a common cause of groin discomfort and pain, and they have been identified in 8–51% of patients undergoing hip arthroscopy. Currently, in the literature there exist three arthroscopic classifications for LT injuries and tears: the first classification was established by Gray and Villar, Botser and Domb proposed the second one which they called a descriptive classification according to the degree of partial thickness LT tears and more recently the last classification by Cerezal *et al.* (*RadioGraphics* 2010; **30**:1637–51), where they take into account the one by Gray and Villar but adding an avulsion fracture and absence of the LT. We propose a new classification, which also takes into account, observed LT pathologies, as well as the possible pathological mechanism of LT tears, and offer a guide to treatment. This classification is based on direct arthroscopic observation and dynamic rotational maneuvers of the hip under distraction. This classification incorporates those pathologies, which have been observed as a result of this more focused examination of the LT.

## INTRODUCTION

**Fig. 1.**
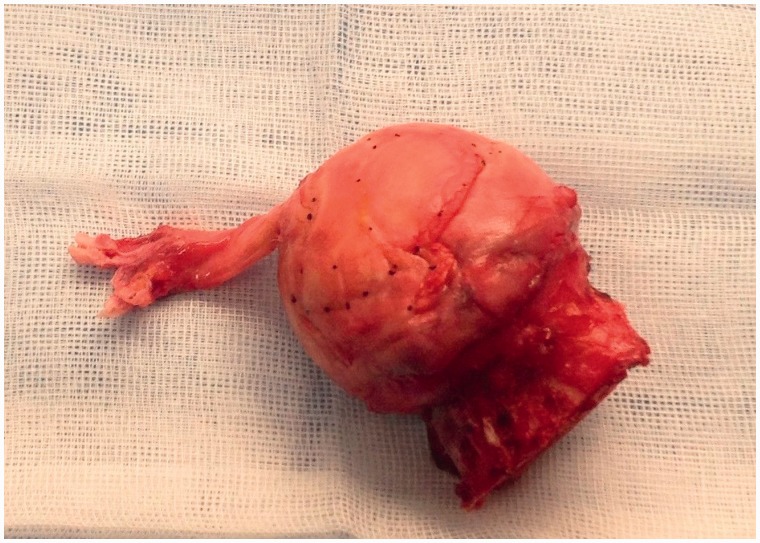
Hip specimen showing the ligamentum of teres and its two bundles.

The ligamentum teres (LT), also known as the round ligament and the ligamentum capitis femoris, is a complex and not a well-understood intra articular structure in the hip joint (Fig. 1). Controversy still exists in regard to the function, of the ligament. It has been suggested that the LT is tightest in the erect position, and that it may have a role in maintaining the upright position [1]; that it participates in fine coordination; that it will function as a strong hip stabilizer specifically in patients with dysplasia, generalized ligamentous laxity, and when there is deficiency of the antero-inferior acetabular wall [2–6]; and also that it may act as a synovial fluid distributor maintaining a lubricated joint [7]; but it has also been suggested that it is an embryonic remnant that does not play any significant role in the adult hip [3].

Only limited literature exists demonstrating its possible function and the role it plays, when damaged, as a source of pain and microinstability in the hip.

A full description of the current understanding of the biomechanics of the LT was recently reviewed by O’Donnell *et al*. [8].

Injuries or tears of the LT have been said to be a common cause of groin discomfort and pain and have been identified in 8–51% of patients undergoing hip arthroscopy (HA) [9]. Pain associated with these tears may dramatically impact on activities of daily living and sport activities. Patients who have been found to have LT tears often describe non-specific symptoms of groin, thigh pain; also they sometimes describe catching, popping, locking and giving way [10–13]. There have been no specific clinical tests until the recent description of the LT Test [14]. To perform the LT test, the hip is flexed to ∼70° and abducted ∼30°. This position minimizes the risk of bony or labral impingement. The hip is then fully internally and externally rotated. Pain provocation in either direction represents a positive test. The authors identified that synovitis about the LT, without an identifiable LT tear, could provoke pain, as well as a torn LT, with or without associated synovitis.

Less than 2% of LT tears are diagnosed on preoperative magnetic resonance imaging and magnetic resonance arthrography (MRA) scans [15]. There have been recent reports of improved accuracy using MRA, and Traction MRA [16–18] but HA remains the gold standard in the diagnosis of LT tears [19].

Currently, there exist three arthroscopic classifications for LT injuries and tears. Gray and Villar proposed the first classification. This classification has three grades of tear: type I complete tear, type II partial thickness tear and type III a tear associated with degenerative changes [20].

Botser and Domb proposed what they called a descriptive classification where they divided the partial tears into two groups. Tears were graded into group I a partial LT tear visualized to be of <50% (low grade); group II a partial LT tear of >50 % (high grade); and a group III a full-thickness LT tear [9], and more recently the last classification by Cerezal *et al.*, where they take into account the one by Gray and Villar but adding an avulsion fracture and absence of the LT.

All of these classifications utilize only a limited description of the appearance of the LT, we propose a new classification, which also takes into account, observed LT pathologies; the possible pathological mechanism of LT tears, and offers a guide to treatment.

Neither of the three current classifications attempts to include the possible underlying pathology which caused the tear, nor do they imply any treatment beyond debridement. Neither classification includes LTs which are abnormal, but not torn. And in addition, the great majority of tears have been shown to be partial thickness tears (using Gray and Villar Classification, 21 type 1, 238 type 2 and 25 type 3 in the study by Botser and Domb), and the current classifications do not adequately differentiate tears according to severity, and possible treatments.

This new proposed classification attempts to address these deficits by providing a more detailed description of the tear types, and more closely aligning the different types with possible pathologic causes, and potential treatments. By so doing, the classification will allow better comparison of series from different centers and allow more accurate comparison of treatments and patient outcomes.

## METHODS

### Surgical technique

As the classification relies on arthroscopic assessment of the LT, a brief description of the important factors in surgical technique is included. The senior author performs HA in a lateral position, supported on a McCarthy hip distractor (Innomed, USA) [21]. Surgery is performed under general anesthesia. No muscle relaxants are used, although they may be added. The padded counter traction post is applied against the proximal femur deliberately raising this post 2–4 cm above the non-operated leg. This positioning will minimize any traction force being applied directly to the perineum, and also allow the weight of the body to apply lateral traction to the proximal femur and lift the femoral head away from the acetabular floor. A similar effect can be obtained with supine positioning by shifting the countertraction post laterally against the proximal femur, and tilting the operating table up on the operated leg side. In order to fully assess the LT, it is essential that the femoral head be drawn laterally, as well as distally, away from the floor of the acetabulum.

Two portals are used routinely to assess the LT. These are a midtrochanteric viewing portal, immediately proximal to the midpoint of the greater trochanter and an anterior paratrochanteric, instrument portal situated 3 cm anterior to the anterior border of the femur, in line with the superior border of the greater trochanter.

A 70° arthroscopic lens is used throughout the procedure.

**Fig. 2.**
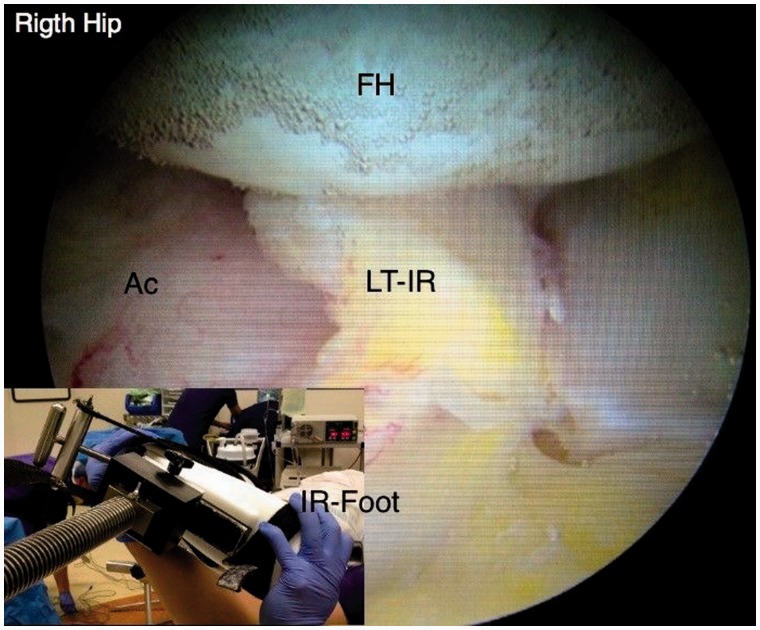
Arthroscopic observation of the LT with the hip in internal rotation, observe the dynamic rotational impingement maneuver under distraction.

**Fig. 3.**
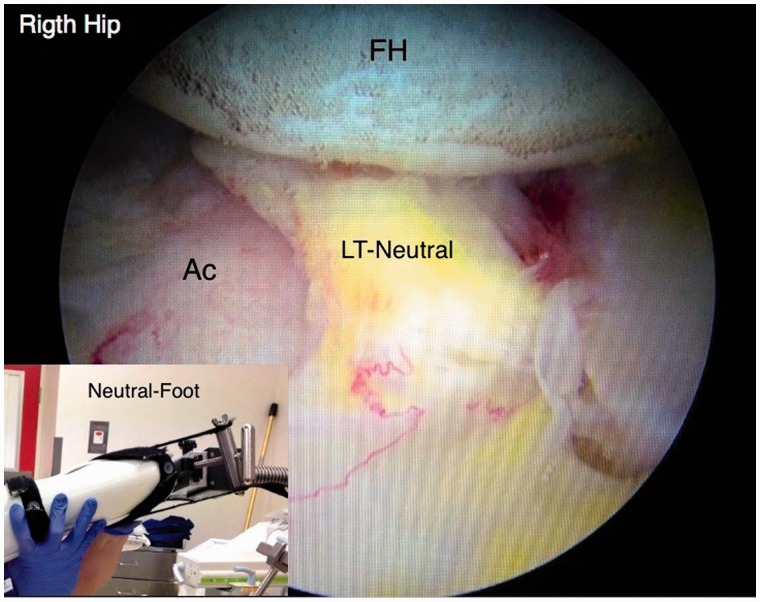
Arthroscopic observation of the LT in neutral position.

**Fig. 4.**
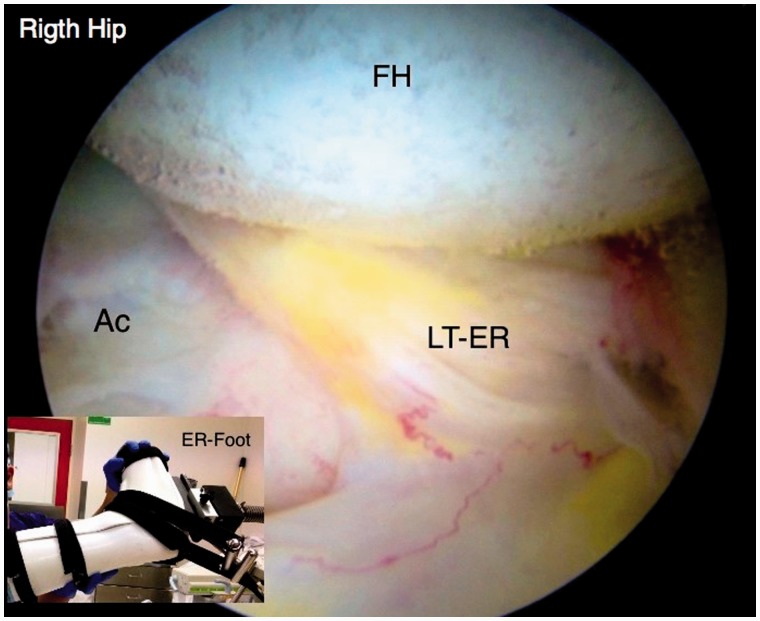
Arthroscopic observation of the LT with the hip in external rotation, observe the dynamic rotational impingement maneuver under distraction.

An initial visual assessment of the LT and adjacent articular cartilage is made. However, full assessment requires also dynamic rotational maneuvers (hip into internal, neutral and external rotation) to examine the behavior of the LT, which may demonstrate tears not otherwise seen (Figs 2–4). This also allows visualization of catching and impingement of the LT against the articular cartilage of the acetabular wall, or bony edge of the acetabular fossa.

**Fig. 5.**
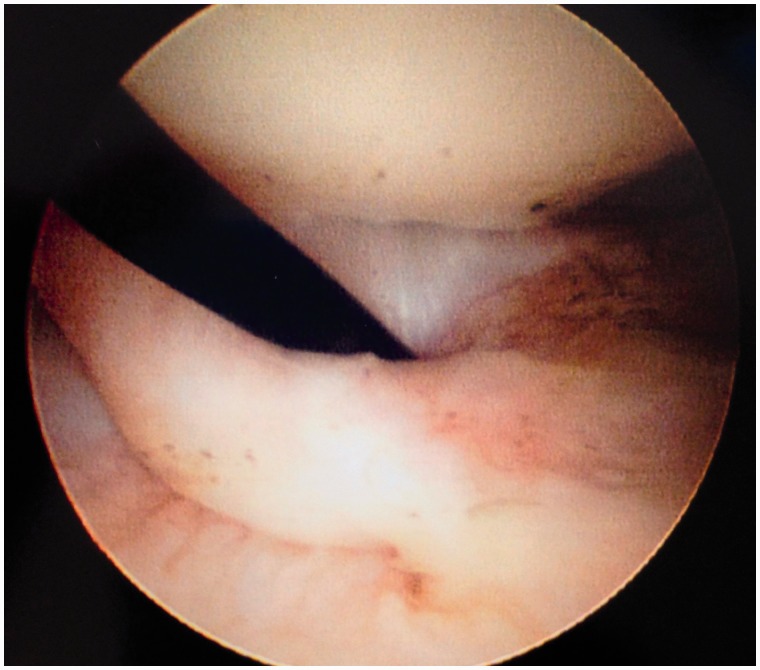
Palpation of the LT with an articulating probe.

Finally, the LT should be probed using bent, or articulating probes (Fig. 5).

## RESULTS

### Classification of LT pathology, treatment proposal and strategies

This classification has built on the classifications of Gray and Villar [20] and Domb and coauthors [9], and has been developed to describe the various LT pathologies observed by the senior author in over 6000 hip arthroscopies. It has also been used to incorporate treatment strategies (TS) (Table I).

**Table I.** Summarized new arthroscopic classification of LT injuries

Type	Description	Treatment proposal/strategy
I. LT synovitis	Inflammation of the synovium of the LT, which may be focal or generalized.	Synovectomy
	Without evidence of impingement (catching or rubbing) on the acetabular wall	
	Redness of the LT, inflamed and bulky synovial tissue (including the acetabular fossa, and pulvinar, as well as the surface of the LT)	
II. LT synovitis with impingement	Inflammation of the LT with impingement on either the articular cartilage of the acetabular wall, or the bony edge of the acetabular fossa	Synovectomy and debridement of the LT
	Impingement against articular cartilage appears as thinning, or color change in the cartilage, and focal hyperemia on the surface of the impinging adjacent LT	Removal of impinging LT ± impinging bone from the fossa edge
III. Partial LT tear—low grade	Flaps of ligament avulsed from the femoral head (most often) or acetabulum	Debridement of LT. RF tightening of LT
	May be associated laxity and hip microinstability	Additional RF, or suture, tightening of the antero-superior capsule to address instability
IV. Partial LT tear—high grade	>50% ligament avulsed from the femoral head or acetabulum or one entire bundle torn or compromised, or a non-functional LT in dynamic rotational maneuvers	Debridement. If required, capsular plication to address instability
V. Partial LT tear associated with *hip osteoarthritis*	Associated with *degenerative changes* of the femoral head and acetabulum, including chondral loss, narrowing of lunate cartilage with calcified spurs, and osteophytes of the acetabular fossa	LT debridement or resection; wash out, removal of floating cartilage pieces, debridement of inflamed synovia, burring of spurs on calcified lunate cartilage
VI. Complete tear of LT	Complete (or near complete) disconnection of LT from the femoral head or acetabulum	Debridement of any residual LT tissue, capsule plication to address instability, consider Ligamentum Teres Reconstruction (LTR) in the unstable hip with normal articular cartilage.
VIa	Acquired	
VIb	Avulsion fracture (non-displaced, partially displaced, or completely displaced)	
VIc	Congenital/absence	

**Fig. 6.**
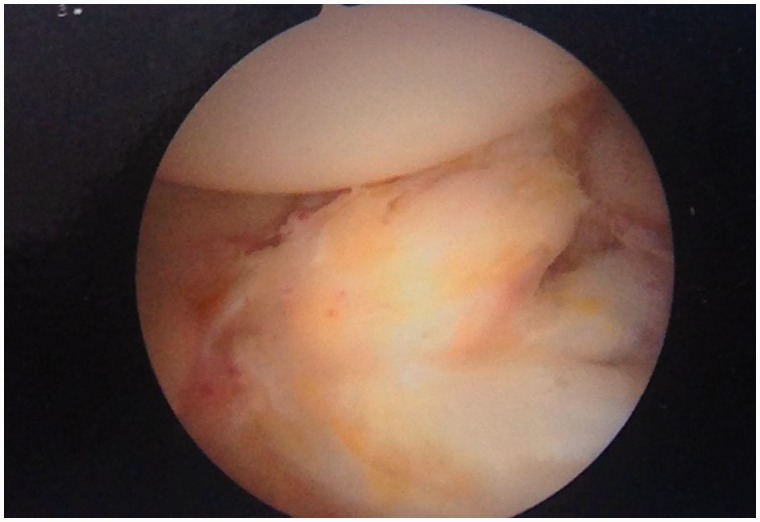
Type I: LT synovitis without impingement (inflammation redness of the LT, inflamed and bulky synovial tissue).

**Fig. 7.**
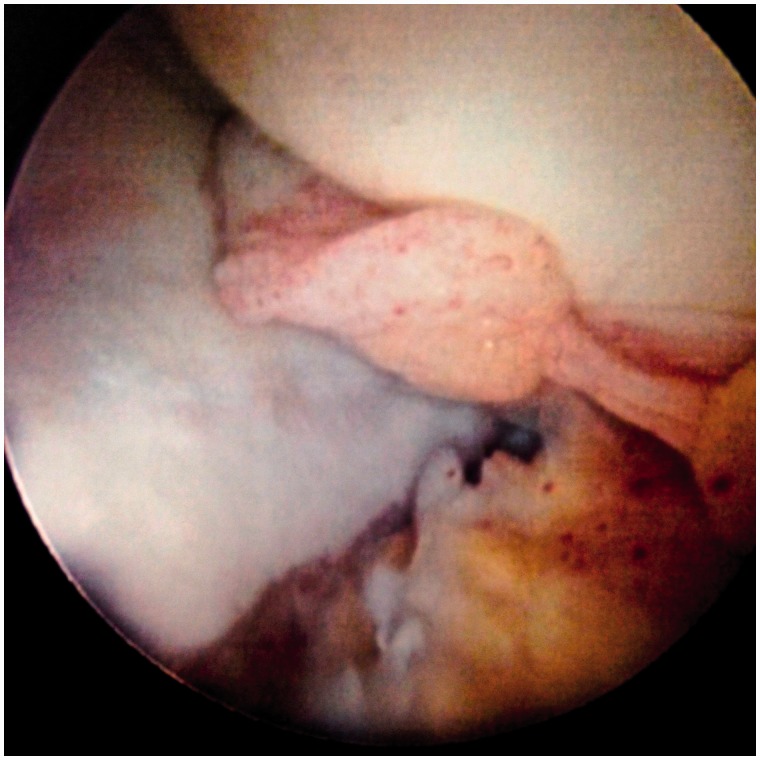
Type II: LT synovitis with impingement. Observe the impingement against the articular cartilage and the focal hyperemia on the acetabular surface.

**Fig. 8.**
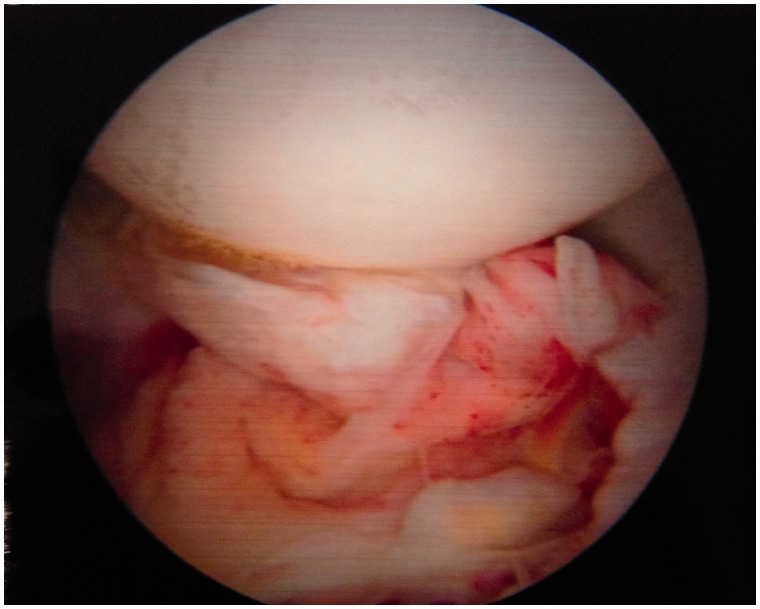
Type III: Partial LT tear—low grade, flaps of ligament avulsed from the femoral head or acetabulum.

**Fig. 9.**
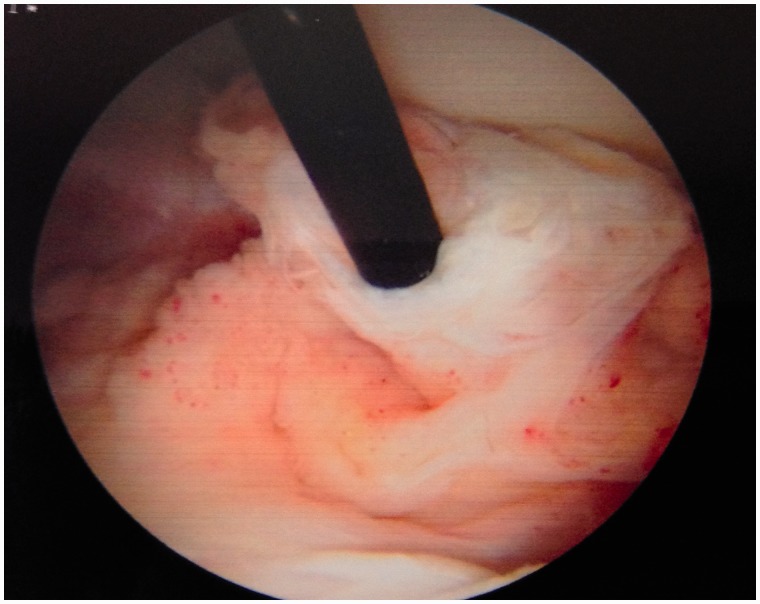
Type IV: Partial LT tear—high grade, one entire bundle torn or compromise (>50% of the torn).

**Fig. 10.**
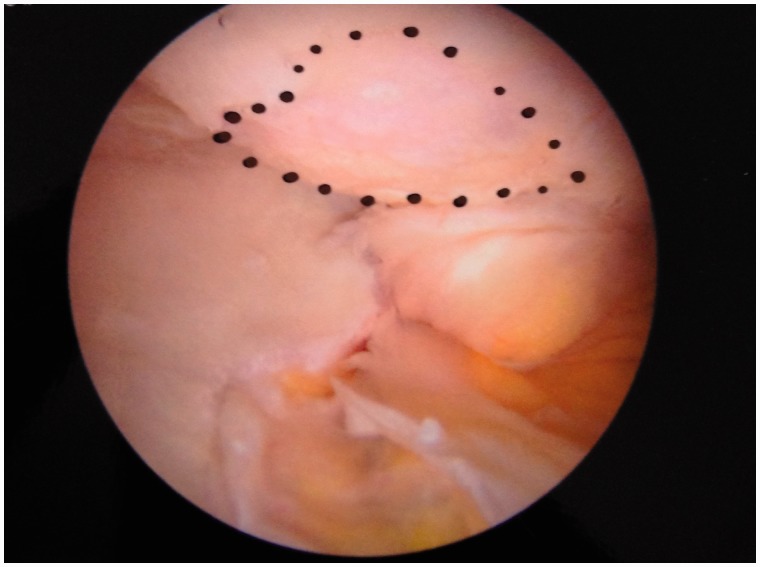
Type V: Partial LT tear associated with degenerative changes.

**Fig. 11.**
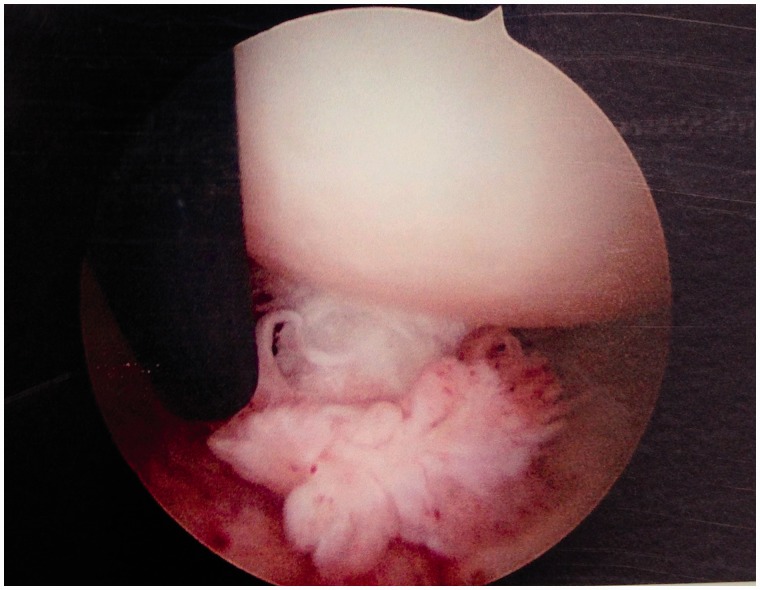
Type VI: Complete (or near complete) LT tear, disconnection of LT from the femoral head or acetabulum.

Figures 6–11 demonstrating the new classification by direct arthroscopic observation of the hip under distraction.

if there is an associated bony problem, e.g. acetabular dysplasia, this will also require treatment on its merits.

Type 1—The LT is intact, and there is associated synovitis, either focal or general about the LT. No apparent cause for the synovitis is identifiable.

### TS—Synovectomy

Type 2—The LT is intact, and there is associated synovitis which matches with a site of apparent impingement. The inflamed area of the LT may be seen to correspond to an area of damaged acetabular articular cartilage [22] or to an area of the bony edge of the acetabular fossa [8].

TS—Synovectomy and debridement of the LT, removal of impinging LT and bone from the fossa edge.

Type 3—Partial tear of LT /low grade. (This corresponds to Domb’s type 1.) The tear is estimated to involve <50% of LT, and in the majority of cases is likely to involve much <50% of LT. There may be an avulsed fragment of fibrocartilage or bone attached to the torn LT.

TS—Debridement of LT, radiofrequency (RF) tightening of LT, additional RF or suture, tightening of the antero-superior capsule to address instability.

Type 4—Partial tear of LT/high grade. (This corresponds to Domb’s type 2.) The tear involves >50% of LT and will generally involve the great majority of LT. There may be an avulsed fragment of fibrocartilage attached to the torn LT.

TS—Debridement of the torn LT. If required, capsular plication to address instability.

Type 5—Partial tear of LT associated with degenerative change of the hip. (This corresponds to Gray and Villar’s type 3.) There will generally be marked synovitis of the LT and pulvinar fat pad associated with is type. As well as loss of acetabular and/or femoral articular cartilage adjacent to the LT, there will often also be osteophytes around the edge of the acetabular fossa.

TS—LT debridement or resection, wash out, removal of floating cartilage pieces, debridement of inflamed synovia, burring of spurs on calcified lunate cartilage.

Type 6—Complete tear, which is divided in 6a Acquired, 6b avulsion fracture of the LT and 6c absence/congenital (this corresponds to Cerezal *et al*. classification).

TS—Debridement of any residual LT tissue, capsule plication to address instability, consider ligamentum teres reconstruction (LTR) in the unstable hip with normal articular cartilage.

## DISCUSSION

Interest in the function and role of the LT and the understanding of the mechanism of injury and tears has increased tremendously in the last few years.

The incidence of LT tears observed at HA appears to have increased substantially also [10, 13]. The reasons for this are not clear, but may include increased awareness of LT tears, and more careful attention to assessment of the LT. There may also be differences in interpretation of what constitutes an LT tear, particularly smaller partial thickness tears.

It has also become apparent that the currently available classification systems for LT tears do not fully incorporate the various pathologies now identified and treated [9, 20]. For example, LTs, which appear to be impinging against the bone of the acetabular fossa edge, but have only associated focal synovitis, and no tear, cannot currently be classified. It has been demonstrated that such lesions can be associated with pain, and treatment by debridement synovectomy and osteectomy of the impinging bone can lead to symptomatic improvement [8].

We have, therefore, proposed a new and more descriptive LT tear classification based on direct arthroscopic observation and dynamic rotational maneuvers of the hip. These movements allow visualization of some tears, which had not been identified during static observation, and also allow identification of areas of LT impingement against bone or articular cartilage. This classification incorporates those pathologies, which have been observed as a result of this more focused examination of the LT.

Hip instability has also been proposed as a cause of LT tear [11, 12]. However, hip instability may be associated with, impingement, partial or complete tears. We have, therefore, not attempted to include an additional classification for instability, but have elected instead to continue to use the appropriate type according to the degree of tear.

We believe that an exhaustive examination which aims to reproduce, as closely as possible natural hip movements (while acknowledging the obvious limitations due to distraction) by the dynamic rotational impingement maneuvers, is mandatory in every HA and patient with suspicion of an LT injury or tear.

### Limitations

This article presents a proposal for a more detailed classification of LT tears, which might be used to help suggest possible treatment options. The proposal is aimed to stimulate debate and lead to an improved LT injury classification. The proposed classification has not yet been validated for use, although we note that the two current classifications have not been validated either. This work remains to be done.

## CONCLUSION

A new classification for LT tears has been proposed to build on the previous classifications of Gray and Villar, and Domb and coauthors, and incorporate new observations of LT pathologies and mechanisms of injury, which will allow more detailed comparison of LT tear treatments and outcomes.

## CONFLICT OF INTEREST STATEMENT

None declared.
